# Gene therapy for ultrarare diseases: a geneticist’s perspective

**DOI:** 10.1186/s12929-024-01070-1

**Published:** 2024-08-13

**Authors:** Wuh-Liang Hwu

**Affiliations:** 1 Center for Precision Medicine, China Medical University Hospital, Taichung City, Taiwan; 2 Department of Pediatrics and Medical Genetics, National Taiwan University Hospital, Taipei City, Taiwan

**Keywords:** Gene therapy, Ultrarare, Lentiviral vector, Adeno-associated viral vector

## Abstract

Gene therapy has made considerable strides in recent years. More than 4000 protein-coding genes have been implicated in more than 6000 genetic diseases; next-generation sequencing has dramatically revolutionized the diagnosis of genetic diseases. Most genetic diseases are considered very rare or ultrarare, defined here as having fewer than 1:100,000 cases, but only one of the 12 approved gene therapies (excluding RNA therapies) targets an ultrarare disease. This article explores three gene supplementation therapy approaches suitable for various rare genetic diseases: lentiviral vector-modified autologous CD34^+^ hematopoietic stem cell transplantation, systemic delivery of adeno-associated virus (AAV) vectors to the liver, and local AAV delivery to the cerebrospinal fluid and brain. Together with RNA therapies, we propose a potential business model for these gene therapies.

## Background

Diseases with low prevalence or incidence, often referred to as rare diseases, place a substantial burden on both health systems and patients. Diagnosing rare diseases is difficult, as physicians are less familiar with rare diseases and their diagnosis sometimes require specialized tests or examinations. Drug development for these conditions also lags behind that for more common diseases. Therefore, to address this issue, many countries encourage the development of orphan drugs by streamlining the approval process and granting sale exclusivity. In the United States, a rare disease is defined as a disease affecting fewer than 20,000 patients. This category includes many genetic diseases and specific cancer subtypes, yet drug development for these diseases remains arduous. However, the rapid advancement of gene therapy in recent years has offered new hope for treating rare genetic diseases. In diseases caused by gene mutations and the resultant loss of gene products, gene therapy aims to treat these conditions by fixing or supplementing the deficient gene. Although the human genome comprises approximately 20,000 protein-coding genes, with mutations in more than 4000 of which are known to cause more than 6000 genetic diseases (OMIM Entry Statistics), the advent of next-generation sequencing (NGS) has significantly enhanced the diagnosis of these diseases, irrespective of their rarity.

Despite these advances, the costs of orphan drug development remain prohibitively high. Consequently, the development and approval of drugs targeting genetic diseases, particularly those affecting very few patients or considered ultrarare, are lacking. Currently, there is no legal definition for “ultrarare” disease; this subcategory was informally introduced by the National Institute for Health and Care Excellence for drugs indicated for diseases with a prevalence of less than 1 per 50,000 people [1]. Some diseases are so rare, with fewer than 30 affected individuals worldwide, that single-subject trials are considered by the n-Lorem Foundation [2]. In this article, we adopt an arbitrary definition of an ultrarare disease as having a prevalence of less than 1 in 100,000 people.

## Current success in gene therapy

Although initial attempts at human gene therapy were met with complications and failures, there has been an increase in the number of approved gene therapy products in recent years [3, 4]. Below are lists of US- and/or EU-approved gene therapies, including RNA therapies, adapted and updated from “The state of cell and gene therapy in 2023” (Tables 1 and 2) [5]. These lists exclude cancer gene therapies.

**Table 1:** List of US- and/or EU-approved gene therapies

No	Product name	Generic name	Company that developed the product	Modality	Disease	Year first approved
1	Strimvelis	Autologous CD34 + enriched cells	GSK	Genetically modified autologous CD34 + HSPCs	Adenosine deaminase deficiency	2016
2	Luxturna	Voretigene neparvovec	Roche	AAV2 gene therapy	Leber's congenital amaurosis	2017
3	Zolgensma	Onasemnogene abeparvovec	Regenxbio	AAV9 gene therapy	Spinal muscular atrophy	2018
4	Libmeldy	Atidarsagene autotemcel	GSK	Genetically modified autologous CD34 + HSPCs	Metachromatic leukodystrophy	2020
5	Skysona	Elivaldogene autotemcel	Bluebird-Bio	Genetically modified autologous CD34 + HSPCs	Adrenoleukodystrophy	2021
6	Upstaza	Eladocagene exuparvovec	PTC-Therapeutics	AAV2 gene therapy	Aromatic L-amino acid decarboxylase deficiency	2022
7	Roctavian	Valoctocogene roxaparvovec	BioMarin	AAV5 gene therapy	Hemophilia A	2022
8	Hemgenix	Etranacogene dezaparvovec	uniQure	AAV5 gene therapy	Hemophilia B	2022
9	Vyjuvek	Beremagene geperpavec	Krystal Biotech	HSV-1 gene therapy	Epidermolysis bullosa	2023
10	Lyfgenia	Lovotibeglogene autotemcel	Bluebird bio	Genetically modified autologous CD34 + HSPCs	Sickle cell anemia	2023
11	Casgevy	Exagamglogene autotemcel	CRISPR Therapeutics	Genetically modified autologous CD34 + HSPCs	Sickle cell anemia	2023
12	Elevidys	Delandistrogene moxeparvovec-rokl	Sarepta Therapeutics	AAVrh74 gene therapy	Duchenne muscular dystrophy	2023

**Table 2:** List of US- and/or EU-approved RNA therapies

No	Product name	Generic name	Company that developed the product	Modality	Disease	Year first approved
1	Kynamro	Mipomersen sodium	Ionis Pharmaceuticals	Antisense therapy	Homozygous familial hypercholesterolemia	2013
2	Exondys 51	Eteplirsen	Sarepta Therapeutics	Antisense therapy	Duchenne muscular dystrophy	2016
3	Spinraza	Nusinersen	Ionis Pharmaceuticals	Antisense therapy	Spinal muscular atrophy	2016
4	Tegsedi	Inotersen	Ionis Pharmaceuticals	Antisense therapy	TTR-related hereditary amyloidosis	2018
5	Onpattro	Patisiran	Alnylam	RNAi	TTR-related hereditary amyloidosis	2018
6	Givlaari	Givosiran	Alnylam	RNAi	Porphyria	219
7	Vyondys 53	Golodirsen	Sarepta Therapeutics	Antisense therapy	Duchenne muscular dystrophy	2019
8	Viltepso	Viltolarsen	Nippon Shinyaku	Antisense therapy	Duchenne muscular dystrophy	2019
9	Waylivra	Volanesorsen	Ionis Pharmaceuticals	Antisense therapy	Lipoprotein lipase deficiency	2019
10	Amondys 45	Casimersen	Sarepta Therapeutics	Antisense therapy	Duchenne muscular dystrophy	2020
11	Leqvio	Inclisiran	Alnylam	RNAi	Heterozygous familial hypercholesterolemia	2020
12	Oxlumo	Lumasiran	Alnylam	RNAi	Hyperoxaluria	2020
13	Nulibry	Fosdenopterin	Orphatec	Oligonucleotide-derived therapy	Molybdenum cofactor deficiency	2021
14	Amvuttra	Vutrisiran	Alnylam	RNAi	TTR-related hereditary amyloidosis	2022
15	Qalsody	Tofersen	Ionis Pharmaceuticals	Antisense therapy	Amyotrophic lateral sclerosis	2023
16	Wainua	Eplontersen	Ionic Pharmaceuticals	Antisense therapy	TTR -related hereditary amyloidosis	2023
17	Rivfloza	Nedosiran	Dicerna Pharmaceuticals	RNAi	Hyperoxaluria	2023

### Approved gene therapies

Luxturna, approved in 2017 for Leber’s congenital amaurosis (LCA), has been a remarkable success, restoring vision in a manner often described as miraculous that subretinal injection of a recombinant adeno-associated virus (AAV) delivering the normal copy of the human RPE65 cDNA led to reversal of blindness [6]. AAV depends on a helper virus to complete its life cycle and does not cause any known human diseases, and AAV rarely integrates into the host genome [7]. This breakthrough underscores the potential of gene therapy to treat previously untreatable diseases. The success of Luxturna encouraged advancements in the field of gene therapy. Following Luxturna, Zolgensma, utilizing an AAV9 virus capsid, was an even greater success for gene therapy [8, 9]. Spinal muscular atrophy (SMA) is an excruciating degenerative disease in which the most severely affected infants do not develop the ability to sit, and others progressively lose motor and respiratory functions. SMA is a relatively common genetic disease, with an incidence of approximately 1 in 10,000 people, creating a substantial market for Zolgensma. From 2019 to 2021, three gene therapies using genetically (lentiviral vector) modified autologous CD34^+^ hematopoietic stem cell transplantation were approved: Zynteglo for β-thalassemia [10], Skysona for adrenoleukodystrophy (ALD) [11, 12], and Libmeldy for metachromatic leukodystrophy (MLD) [13]. β-Thalassemia and ALD can also be treated with allogeneic hematopoietic stem cell transplantation (HSCT), but gene therapy offers a viable alternative when a suitable donor is unavailable, and autologous transplantation presents a lower risk than allogeneic transplantation. In 2022, Upstaza, rAAV2-hAADC, was approved for treating aromatic l-amino acid decarboxylase (AADC) deficiency, marking the first gene therapy targeting the brain directly. The same year, Roctavian and Hemgenix were approved for treating hemophilia A [14] and B [15], respectively. Gene therapy for hemophilia has achieved success comparable to that of Zolgensma, driven by the relatively high prevalence of hemophilia (hemophilia A affects 1 in 5,617 males, and hemophilia B affects 1 in 19,283 males). This success is particularly important considering the expensive and inconvenient nature of coagulation factor infusions. In 2023, four more gene therapies were approved: Vyjuvek for epidermolysis bullosa [16]; Lyfgenia, which uses a lentiviral vector encoding the antisickling hemoglobin HbA^T87Q^, for sickle cell anemia [17]; Casgevy, which employs CRISPR-Cas9, the first gene editing therapy, to target the *BCL11A* erythroid-specific enhancer [18], also for sickle cell anemia; and Elevidys, which is used for Duchenne muscular dystrophy (DMD) [19], a genetic muscular degenerative disease affecting approximately 1 in 5,000 males.

### Approved RNA therapies

There are more approved RNA therapies than gene supplementation therapies (Table 2). Antisense oligonucleotides (ASOs) and small interfering RNAs (RNAi) are two widely used strategies for silencing gene expression [20]. Companies such as Ionis Pharmaceuticals and Sarepta Therapeutics have developed several antisense therapies for Duchenne muscular dystrophy, transthyretin (TTR)-related hereditary amyloidosis, and spinal muscular atrophy (Table 2). Companies such as Alnylam developed RNAi therapies for TTR-related hereditary amyloidosis, porphyria, hyperoxaluria, etc. (Table 2). TTR-related amyloidosis is caused by systemic deposition of transthyretin, with clinical manifestations including neuropathy, cardiomyopathy, and oculoleptomeningeal involvement [21]. ASO and RNAi are highly effective at disrupting complementary mRNAs and inhibiting TTR synthesis. Since their development, Tegsedi (antisense therapy), Onpattro (RNAi), Amvuttra (RNAi), and Wainua (antisense therapy) have all been licensed for the treatment of TTR-related hereditary amyloidosis [22].

### Clinical trials

There are also clinical trials for new treatments. Liver transduction of AAV-G6PC increases the long-term efficacy of treatment for glycogen storage disease type Ia [23]. Gene therapies with different strategies, including liver depot gene therapy, have been tested for the treatment of glycogen storage disease type II (Pompe disease) [24, 25]. More genetic diseases of the eyes are being investigated. Treatment for patients with ABCA4-associated Stargardt disease is conducted with an equine infectious anemia virus-driven vector (EIAV-ABCA4) [26]. Antisense oligonucleotides rescue aberrant splicing caused by an ultrarare ABCA4 variant in children with early-onset Stargardt disease [27]. AAV5-NR2E3 may attenuate retinal degeneration caused by rhodopsin and other gene mutations in patients with retinitis pigmentosa [28]. Moreover, a single administration of lipid nanoparticles loaded with gene editor mRNAs could inactivate the Pcsk9 gene to treat genetic and acquired hypercholesterolaemia [19], and currently, the heart-1 study is ongoing [29].

### Rare diseases having intense drug developments

Rare diseases, such as DMD, have been approached by different ways [30, 31]. Around 80% of DMD mutations are potentially amenable to exon skipping. Eteplirsen (Exondys 51, Sarepta Pharmaceuticals) was the first exon-skipping pharmacologic treatment approved by the FDA in 2016 [32]. Exon 53 skipping, golodirsen (Vyondys 53, Sarepta Pharmaceuticals), increases the proportion of eligible DMD patients by a few percent [33]. The readthrough drug ataluren (Translarna) targets approximately 13% of DMD patients who have a nonsense mutation [34]. Moreover, Elevidys, an AAV-based gene supplementation therapy, supplies a copy of microdystrophin cDNA that could benefit all DMD patients [35].

Another example is Huntington’s disease (HD) which is caused by a pathological expansion of CAG repeat on the huntingtin gene. There are several ways to decrease the expression of the mutant Huntingtin protein [36, 37]. Huntingtin suppression with ASOs specific to HD mutation linked single-nucleotide polymorphisms restores cognitive function in a mouse model of HD [38]. Mutant huntingtin lowering ASOs can be delivered to the brain through systemic administration using apolipoprotein A-I nanodisks [39]. miRNA can also lower huntingtin levels and preserves striatal volume and cognitive function in a humanized mouse model of HD [40]. An orally available, brain penetrant, small molecule lowers huntingtin levels by enhancing pseudoexon inclusion [41]. Overexpression of sterol regulatory element-binding protein 2 (SREBP2) in HD mice activates the transcription of cholesterol biosynthesis pathway genes, clears mutant huntingtin aggregates, and attenuates behavioral deficits [42].

### Few treatments for ultrarare genetic diseases

Among these diseases for which gene therapy has been approved, SMA, DMD, and hemophilia A have the highest incidence, occurring in at least 1 in 10,000 individuals. The incidence of LCA ranges from 1 in 33,000 to 80,000. AADC deficiency, with only 140 documented cases (Orphanet Report November 2023), stands out as the sole ultrarare disease in this context. ALD (1:20,000–50,000) and MLD (1:40,000–100,000) have higher incidences than AADC deficiency; however, only a subset of patients are eligible for gene therapy, which must be administered before symptoms manifest. ALD can also be treated with allogeneic HSCT, which might pose challenges for Bluebird Bio to profit from these two products. Moreover, substantially more resources are being dedicated to gene therapy for cancer than for genetic diseases, particularly chimeric antigen receptor (CAR) T-cell therapy [43]. RNA therapy has also achieved several successes, but its high application potential lies in the ability of its production to be scaled up to treat a large number of patients. Consequently, developing gene therapy for ultrarare genetic diseases often faces difficulty in securing funding or resources.

## Burden of ultrarare genetic disease

Given the vast number of genetic diseases, many geneticists routinely encounter a wide variety of conditions in practice. An experienced geneticist can typically identify hundreds of genetic diseases. For instance, mucopolysaccharidoses (MPSs) encompass types I, II, III, IV, VI, and VII. Mucolipidoses (MLs) include sialidosis, galactosialidosis, MLII, and MLIII. Glycolipidoses include Gaucher disease, Niemann-Pick A/B, Niemann-Pick C, GM1 and GM2 gangliosidosis, and Fabry disease. Glycogenosis encompasses glycogen storage disease types IA, IB, II (Pompe disease), and III. Neurotransmitter deficiency includes AADC deficiency, deficiency of tyrosine hydroxylase (TH), deficiency of 6-pyruvoyl-tetrahydropterin synthase (PTPS), and deficiency of GTP cyclohydrolase 1 (GCH1). Other metabolic diseases include urea cycle disorders, aminoacidopathies, and organic acidurias. Skeletal diseases include Ehlers–Danos syndrome (EDS), spondyloepiphyseal dysplasia (SED), and osteogenesis imperfectia (OI). Additional categories include congenital generalized lipodystrophy, diseases involving DNA repair defects, and various etiologies of early infantile epileptic encephalopathy (EIEE). Recently, we described the genetic etiology of 34 patients with skeletal diseases, identifying 16 genes involved in these conditions [44], with an average of two patients per disease. During my 30-year practice, I have seen all the diseases mentioned here. Regrettably, few specific treatments are available, and among them, AADC deficiency is the only ultrarare disease for which gene therapy has been developed [45]. With sufficient resources, many of these conditions can be treated with gene therapy.

## Gene therapy modalities suitable for treating ultrarare genetic disease (Fig. 1)

**Fig. 1:**
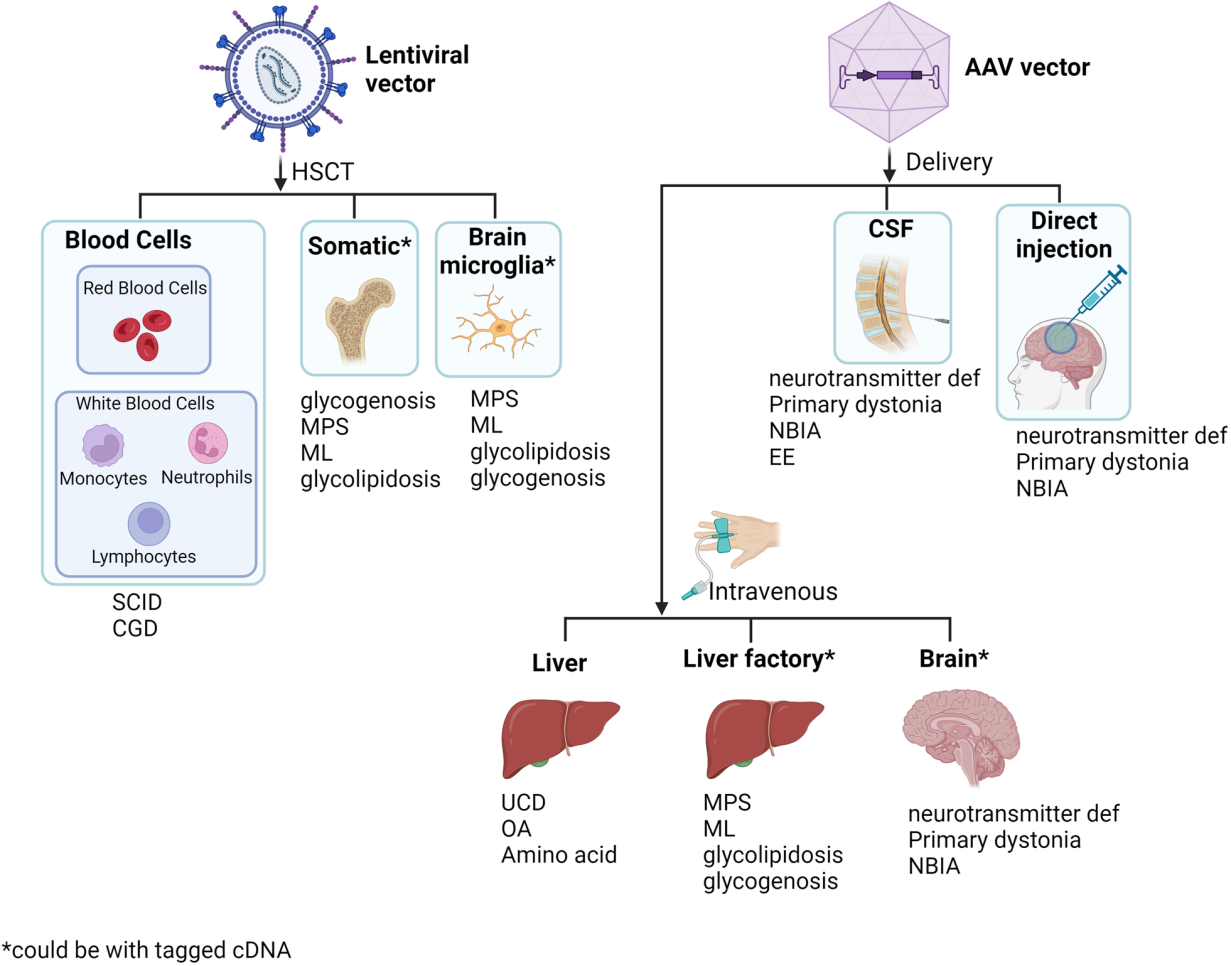
Application of gene therapy for ultrarare diseases. *CGD* chronic granulomatous disease, *EDS* Ehlers–Danos syndrome, *EE* epileptic encephalopathy, *ML* mucolipidoses, *MPS* mucopolysaccharidoses, *NBIA* neurodegeneration with brain iron accumulation, *OA* organic acidurias, *OI* osteogenesis imperfecta, *SCID* severe combined immunodeficiency, *SED* spondyloepiphyseal dysplasia, neurotransmitter deficiency, *UCD* urea cycle disorders

### Lentiviral vector-modified autologous CD34^+^ hematopoietic stem cell transplantation [46] (Fig. 2)

**Fig. 2:**
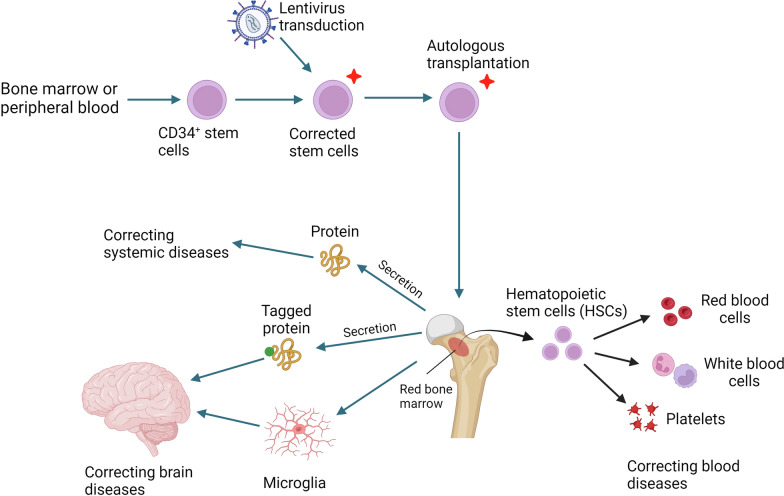
Mechanisms of lentiviral vector-modified autologous CD34^+^ hematopoietic stem cell transplantation. Transduction of hematopoietic stem cells can correct functional defects in blood cells, including T cells, B cells, and macrophages. Transgenes can encode secretory proteins that are secreted into the systemic circulation. The secreted protein, if it contains a brain-targeting epitope, can enter the brain. Bone marrow-derived cells can also migrate to the brain and differentiate into microglia, which can ameliorate brain defects

Among the 12 previously mentioned approved gene therapies for genetic diseases, six utilize lentiviral vector-modified HSCT for patients with adenosine deaminase deficiency, β-thalassemia, MLD, ALD, and two distinct products for sickle cell anemia. Lentiviral vectors, such as those used to treat thalassemia, are capable of delivering complex tissue-specific expression cassettes to nondividing cells [10] without promoting leukemogenesis, in contrast to the γ-retroviral vectors used in earlier clinical studies [47]. In theory, any genetic disease treatable by traditional allogeneic HSCT could also benefit from lentiviral vector-modified HSCT; moreover, autologous transplantation in gene therapy is considered safer than allogeneic transplantation. Allogeneic HSCT has proven effective for treating diseases such as Gaucher disease and MPSI, although enzyme replacement therapy (ERT) is preferred due to safety considerations. However, in diseases affecting the brain, such as Gaucher disease type III and MPSIH/S, allogeneic HSCT is still considered a treatment option [48]. It is understood that transplanted hematopoietic stem cells can differentiate into phagocytic cells that migrate to the brain as microglia [49], which can then clear abnormal metabolites in diseases such as ALD or secrete molecules to rescue host brain cells in lysosomal storage diseases.

The advantages of lentiviral vector-modified HSCT over allogeneic HSCT also include the use of stronger promoters to increase product expression and codon optimization to increase translation efficiency. Genetic engineering can add a secretory leader peptide to the gene, achieving high blood levels of the transgene product akin to ERT. Lentiviral vector-modified HSCT can maintain high and stable blood levels of the expressed protein, unlike the pulsatile blood protein levels observed in ERT. Recently, brain-targeted ERT, developed by adding brain-penetrating epitopes to the infused protein [50], such as a transferrin epitope or an antibody to the receptor on the infused protein, has been shown to facilitate transferrin receptor-mediated transport across the blood–brain barrier. This strategy can be easily adapted to lentiviral vector-modified HSCT by modifying the cDNA sequence in the vector [51].

However, lentiviral vector-modified HSCT has limitations and complications. Currently, it is impossible to regulate the expression level of the transgene after viral vector transduction. While the overexpression of the transgene may be tolerable in conditions such as lysosomal storage diseases, where treatment necessitates the delivery of large amounts of enzyme systemically or to the cerebrospinal fluid (CSF), it can be toxic in other contexts. For instance, in gene therapy for AADC deficiency, the enzyme responsible for monoamine neurotransmitter production is delivered directly to the putamen to prevent ectopic or excessive dopamine production in the brain. Similarly, overexpression of β-globin in therapies for β-thalassemia could lead to a relative deficiency of α-globin, essentially converting the disease to α-thalassemia. Thus, the lentiviral vector used for gene therapy of β-thalassemia contains regulatory elements for the β-globin gene. While lentiviral vectors have been proven to be safer than retroviral vectors and are not associated with vector insertion-related leukemia, they have recently been suspected to cause myelodysplasia (MDS) [52]. Given the novelty of gene therapy, a careful assessment of risks and benefits is necessary before proceeding with treatment.

### Systemic delivery of AAV vector to the liver (Fig. 3)

**Fig. 3:**
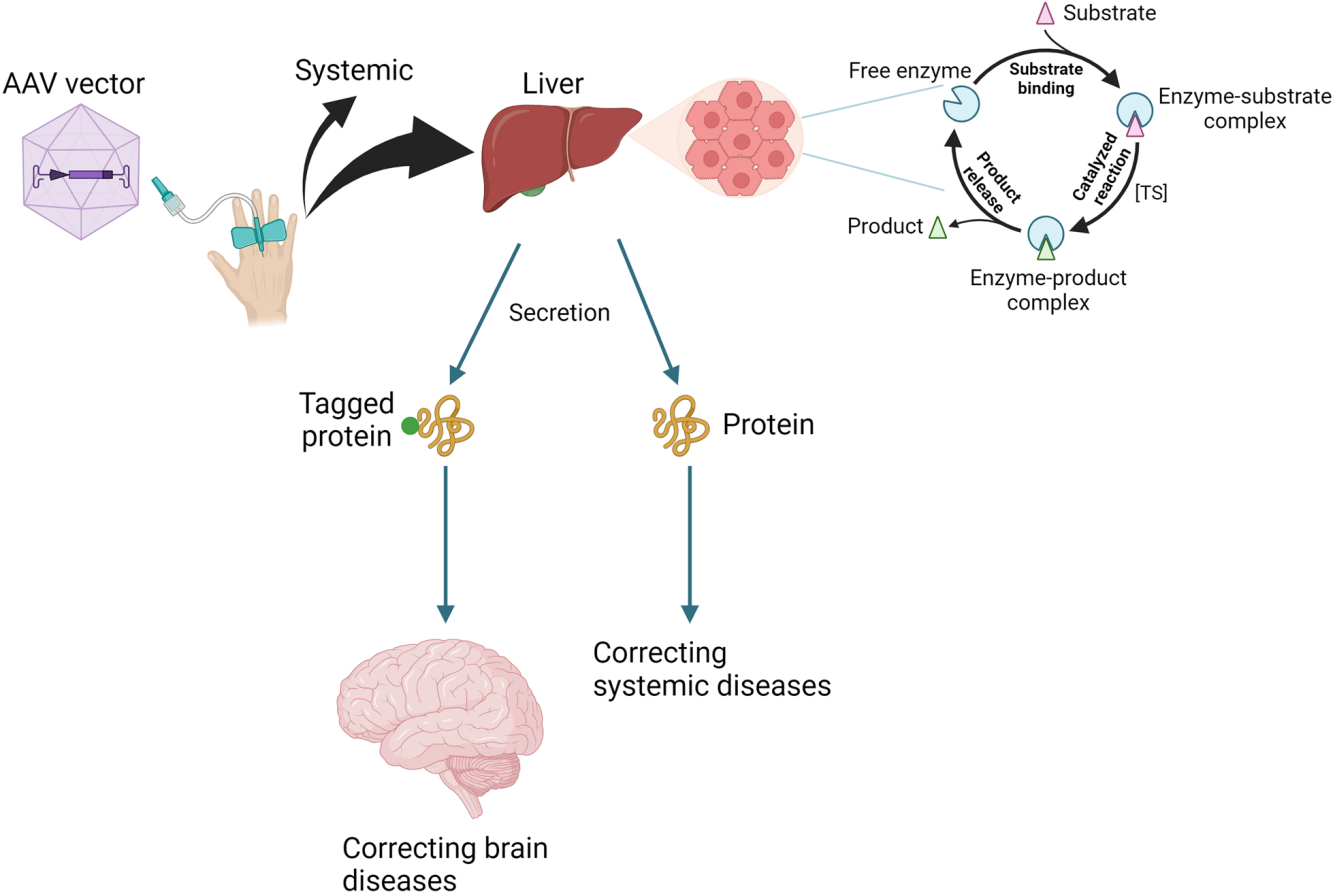
Mechanism of systemic AAV vector delivery to the liver. After intravenous infusion, the majority of AAV vectors are taken up by the liver. The transduced hepatocytes restore their functions, such as by conducting an enzyme reaction. If the transgene encodes a secretory protein, the protein can be released into the circulation to treat systemic diseases. The secretary protein, if it contains a brain-targeting epitope, can enter the brain

Among the 12 approved gene therapies for genetic diseases, two target the liver (hemophilia A and B), one targets the nervous system (SMA), and one targets the muscle (DMD) using systemic delivery of AAV vectors. The systemic infusion of Zolgensma is used to treat children with SMA, but plans are in place to deliver the vector to the CSF in adult patients [53]. Elevidys, recently approved for DMD treatment at a dose of 1.33 × 10^14^ vector genomes per kilogram (vg/kg) of body weight, is still under evaluation for both efficacy and potential adverse effects. Systemic delivery of AAV to muscles requires high doses and is more likely to cause complications, including liver toxicity and microangiopathy [54]. Conversely, since the liver retains more than 90% of systemically infused AAV vectors, systemic delivery to the liver requires only one-tenth the dose needed for muscle targeting, significantly reducing the risk of complications [55]. Valoctocogene roxaparvovec (AAV5-hFVIII-SQ) contains a coagulation factor VIII cDNA driven by a liver-selective promoter [14]. In a phase 3 study, 134 participants with hemophilia A received a single infusion of 6 × 10^13^ vg/kg of vector. The mean factor VIII activity level at one year increased by 41.9 IU per deciliter. The mean annualized rates of factor VIII concentrate use and treated bleeding after week 4 decreased after infusion by 98.6% and 83.8%, respectively. There was no mortality in the trial. Etranacogene dezaparvovec is an AAV5 vector expressing the Padua factor IX variant. In a phase 3 study, 54 men with hemophilia B received 2 × 10^13^ genome copies per kilogram of body weight of the vector. The annualized bleeding rate decreased from 4.19 during the lead-in period to 1.51 during months 7 through 18 after treatment [15].

In the case of hemophilia, the liver is pivotal because it produces coagulation factors. Many other genetic diseases, including metabolic diseases such as organic acidurias, urea cycle disorders, and aminoacidopathies, are also caused by liver dysfunction. Like all vector-mediated gene therapy, the efficacy can be enhanced with stronger or more appropriate promoters, codon optimization, secretory leader peptides, and epitopes targeting organs such as the brain. Systemic delivery of the AAV44.9-*Mmut* vector has been shown to prevent lethality and lower disease-related metabolites in methylmalonic acidemia mice. Tissue biodistribution and transgene expression studies in treated mice showed that AAV44.9 was efficient at transducing the liver and heart [56]. Pompe disease is a lysosomal storage disorder causing skeletal muscle weakness and cardiomyopathy. Recent data reveal that 2 × 10^11^ vg/kg of AAV2/8-LSPhGAA, containing a liver-specific promoter/enhancer and a leader sequence, transduced all hepatocytes which led to partial biochemical correction in adult GAA-KO mice with Pompe disease [57].

However, systemic AAV delivery is hindered by preexisting antibodies to the viral capsid, excluding some patients from therapy [58]. Additionally, hepatocyte turnover can lead to a decrease in transgene expression over time, as in most liver-directed gene therapies, although the duration until therapeutic effects diminish remains uncertain [55, 59]. Last, although AAV is not an integrating virus, a small amount of integration into the host genome has been observed [60], and the long-term implications of this viral integration are yet to be fully understood.

### Local delivery of AAV vectors to the CSF and brain for neurological disease treatment (Fig. 4)

**Fig. 4:**
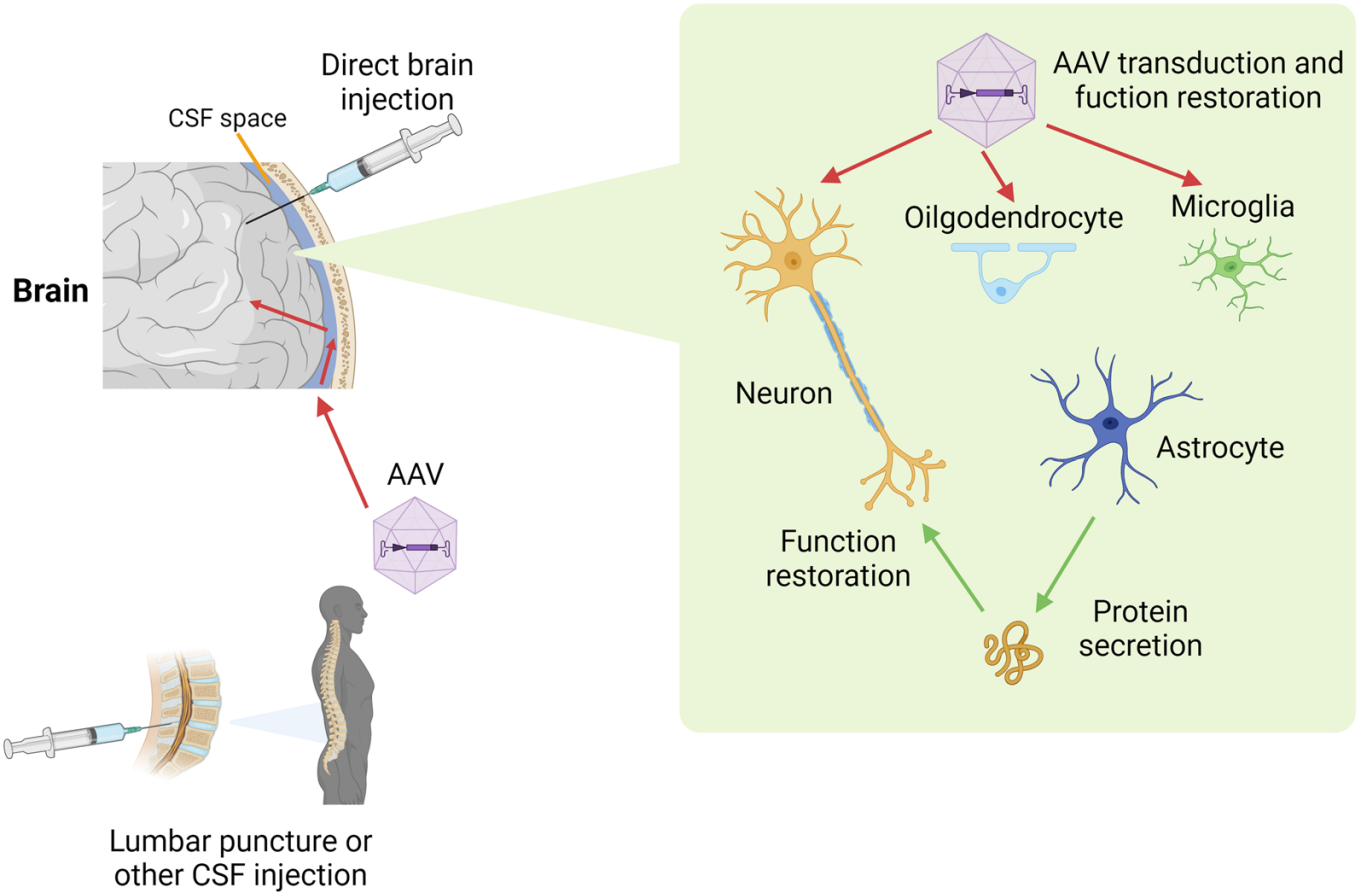
The mechanism of AAV vector delivery to the brain. AAV vectors can be administered directly into the brain parenchyma or injected into CSF spaces (e.g., by lumbar puncture) and the vectors then migrate to the spinal cord and brain. AAVs can transduce neural cells in the brain and ameliorate their dysfunction. Transgenes may be secreted to alleviate the dysfunction of other cells

Targeting is still the greatest challenge in gene therapy. For example, scientists are working on the development of modified AAV vectors that target muscle cells while also reducing liver uptake [61]. Reprogramming of the AAV capsid to mediate brain gene delivery has also been undertaken [62]. However, AAV vectors can be easily administered directly into CSF spaces (intrathecal or intracisternal) or the brain parenchyma to treat diseases affecting the brain, spinal cord, eyes, and ears. CSF and brain delivery have several advantages. First, the vector quantity required is substantially less than that needed for systemic infusion—an order of magnitude less than systemic AAV delivery to the liver. This reduction saves costs and minimizes the risk of systemic complications such as liver damage. Second, the challenge posed by preexisting antibodies is less important for CSF and brain delivery due to the immune privilege of the central nervous system. Third, since neurons do not divide, the therapeutic effects of AAV can be long-lasting. Fourth, delivering the vector directly to the brain reduces the risk of ectopic expression of the transgene.

Among the 12 approved gene therapies for genetic diseases, one targets the eyes (LCA), and one targets the putamen (AADC deficiency) via local injection of AAV vectors. Both utilize AAV2, which exhibits high tropism for neuronal cells and limited tissue distribution. These treatments use minimal amounts of vector and are characterized by high efficacy and a lack of significant complications. Currently, gene therapy is advancing for several eye and ear diseases [63]. Additionally, various brain disorders could benefit from brain-directed AAV injections. Monoamine neurotransmitter deficiencies can be treated by putamen injections [64]. Neurodegeneration with brain iron accumulation (NBIA) could be treated by injection into the putamen or globus pallidus [65]. Primary dystonia may be relieved by targeting the thalamus [66]. The delivery of AAV vectors to CSF spaces is an option for treating diseases with more widespread brain pathologies. Intracerebroventricular AAV delivery in humans predominantly transduces ependymal cells [67]. Delivery through lumbar puncture is both convenient and practical, particularly for spinal cord diseases [68]. Direct injection into the cisterna magna (intracisternal injection) may provide a balanced vector distribution between the brain and spinal cord [68]. These methods could benefit many brain diseases or systemic diseases with brain manifestations, including some lysosomal storage diseases [69].

The primary limitation of brain delivery is whether the vector distribution is sufficiently broad to encompass all affected brain regions. For example, CSF delivery typically leads to the transduction of a small number of neurons in the putamen, while intraputaminal injections do not affect cells in the cortex or cerebellum. Moreover, since brain pathologies in most diseases are not reversible, the potential efficacy of gene therapy can be limited.

### RNA therapy and gene editing for ultrarare disease

The abovementioned three modules of gene therapies are mostly involved in gene supplementation, but RNA therapy is also emerging as one of the most promising treatments for ultrarare diseases [2]. Single-stranded DNA or RNA oligonucleotides bind RNA and block gene expression, modulate splicing, cleave DNA•RNA hybrids via RNase H, and target miRNAs [20]. Double-stranded short interfering RNAs (siRNAs) bind the protein machinery of the RNA-induced silencing complex (RISC), and the RISC directs its bound small RNA to target complementary RNAs and represses their expression through mRNA cleavage, degradation, and translational repression [70]. Modification of DNA or RNA oligonucleotides by phosphorothioate (PS) linkages and chemical modifications greatly improves their stability, binding to serum proteins, and binding affinity for their complementary sequences [71, 72].

ASOs and RNAi can reduce mRNA levels and suppress the expression of mutated toxic gene products, as observed in HD [37], or target upstream or downstream genes in metabolic pathways to mitigate the detrimental effects of mutations, such as acute hepatic porphyria [73]. Additionally, ASOs can regulate mRNA splicing; for instance, in treating DMD, they can splice out exons containing missense or nonsense mutations to restore protein translation, producing a shorter but functional dystrophin protein that alleviates symptoms [30]. In more unique cases, ASOs interfere with the binding of splice suppressors, as in the treatment of SMA, to enable correct splicing [74].

Although RNA therapy drugs require regular administration to treat genetic diseases, oligonucleotides can be produced and purified like small molecule drugs, offering the same advantages of low production costs and scalability. The behaviors of ASOs are quite consistent, resulting in predictable therapeutic doses, routes of administration, frequencies of dosing, and potential side effects [75]. Recently, n-Lorem collaborated with Ionis Pharmaceuticals to discover and develop personalized ASOs for one patient at a time—N-of-1 therapies [76, 77]. The possibilities of ASOs and RNAi therapies for treating monogenic disorders have recently been reviewed [78].

Gene editing, mainly using CRISPR/Cas, represents another powerful tool for effectively disrupting gene expression [79]. Currently, the first approved clinical application of CRISPR/Cas9 is for treating sickle cell anemia. Due to concerns about the risks associated with double-strand breaks [18], base editing, a derivative of CRISPR/Cas9 that modifies bases without inducing double-strand breaks, is a safer alternative that has catalyzed several clinical trials [80]. Nonetheless, the potential for off-target effects [81] necessitates cautious application of base editing in treatments for ultrarare diseases.

In Table 3, we compare the treatment modules mentioned in this article and note the feasibility of applying them to treat ultrarare diseases.

**Table 3:** Comparison of different treatment modules

Treatment module	Advantage	Drawback	Making a new drug for ultrarare disease
Small molecule	Simple oral medication	High cost and slow in developing a new small molecular drug	Difficult
Enzyme replacement therapy	Highly effective	High cost in development and poor brain penetration	Difficult
Lentivirus-hematopoietic stem cell transplantation	Highly effective for hematopoietic disease, produces secretary proteins	Transplantation required, risk of lentiviral integration	Yes, could be applied to multiple ultrarare diseases
Systemic AAV targeting the liver	Highly effective for liver disease, produces secretary proteins	Loss of effect after cell turn over, risk of AAV integration (rare)	Yes, could be applied to multiple ultrarare diseases
Systemic AAV targeting organs other than the liver	Easy intravenous infusion, effective for multiple diseases	High cost of large quantity AAV production, AAV systemic complication, AAV integration	Difficult
local delivery of AAV vectors to the CSF and brain	Convenient for neurological disease, persistent effect	Effectiveness depending on viral distribution in the brain	Yes, could be applied to multiple ultrarare diseases
RNA therapy	Easy to design and produce	Need a suitable mechanism such as gene suppression or splicing	Yes, but need continuous drug administration
Gene editing	Regulates gene expression or correct gene defect	Risk of genotoxicity	Maybe, but not applicable at the present time

## Funding, reimbursement, and business model

Although gene therapy has the potential to transform the lives of people living with these devastating rare diseases, accessing these new therapies is far from straightforward for patients [82]. The obstacles in developing treatments for rare diseases extend beyond technological issues, including funding, reimbursement strategies, and business models. While most currently approved gene therapies target rare diseases, they collectively impose a substantial financial impact on health and insurance systems [83]. This has prompted the proposal of various payment methods and policies to ensure that patients can access the benefits of gene therapy, for example, to increase drug affordability through health care loans [84]. A business model for the treatment of ultrarare diseases is also difficult. One innovative approach suggested by the n-Lorem organization, particularly for ultrarare diseases affecting fewer than 30 people globally, involves treating one patient at a time using a nonprofit model [76].

Nevertheless, the gene therapy modalities discussed in this article—lentiviral vector-modified autologous CD34^+^ hematopoietic stem cell transplantation, systemic delivery of AAV to the liver, delivery of AAV to the CSF and brain, and RNA therapies—have the potential to treat multiple genetic diseases. In a regulatory environment conducive to innovation, a company could specialize in a single technique for multiple diseases, thereby saving on development costs, vector production, biodistribution, and toxicity testing. By partnering with patient groups and medical societies eager to deliver treatments to their patients, clinical trials could be designed more cost-effectively [85]. For example, when we developed Upstaza for AADC deficiency, the first and only brain-directed gene therapy targeting an ultrarare disease, we developed it through academic research grants before transitioning to a commercial setting [86]. These strategic approaches will allow companies to remain profitable while expanding access to treatments for more patients with ultrarare diseases.

## Conclusions

This article overviews current successes in gene therapy, including autologous lentiviral vector-modified hematopoietic stem cell transplantation to treat hematological and neurological diseases and AAV vector-mediated gene therapy to treat eye, liver, and neurological diseases. These new technologies provide hope for the thousands of individuals with rare genetic diseases. However, both the high risk and cost of gene therapy prevent its rapid development, especially for ultrarare diseases with only a small number of eligible patients. In this article, we propose several gene therapy technologies that are suitable for treating rare genetic diseases. For example, in the systemic delivery of AAV vectors, liver targeting requires fewer vectors but can treat both liver and systemic diseases. Direct delivery of the AAV vector to the nervous system can also treat neurotransmitter deficiency, primary dystonia, and NBIA. A company can therefore specialize in one technology that can target multiple ultrarare diseases to decrease the financial burden of gene therapy development. We hope that more patients with ultrarare genetic disease can receive gene therapy soon.

## Data Availability

Not applicable.

## Abbreviations

AADC: Aromatic l-amino acid decarboxylase
AAV: Adeno-associated virus
ALD: Adrenoleukodystrophy
ASO: Antisense oligonucleotide
CAR: Chimeric antigen receptor
CSF: Cerebrospinal fluid
DMD: Duchenne muscular dystrophy
EDS: Ehlers–Danos syndrome
EIEE: Early infantile epileptic encephalopathy
ERT: Enzyme replacement therapy
GCH1: GTP cyclohydrolase 1
HD: Huntington’s disease
HSCT: Hematopoietic stem cell transplantation
LCA: Leber’s congenital amaurosis
ML: Mucolipidoses
MDS: Myelodysplasia
MLD: Metachromatic leukodystrophy
MPS: Mucopolysaccharidoses
NBIA: Neurodegeneration with brain iron accumulation
NGS: Next-generation sequencing
OI: Osteogenesis imperfect
PTPS: 6-Pyruvoyl-tetrahydropterin synthase
RISC: RNA-induced silencing complex
RNAi: Interfering RNA
SED: Spondyloepiphyseal dysplasia
siRNA: Short interfering RNA
SMA: Spinal muscular atrophy
TH: Tyrosine hydroxylase
TTR: Transthyretin

## References

[1] Harari S, Humbert M. Ultra-rare disease: an European perspective. Eur Respir Rev. 2020;29:200195. 32620589 10.1183/16000617.0195-2020 PMC9488651

[2] Crooke ST. Meeting the needs of patients with ultrarare diseases. Trends Mol Med. 2022;28:87–96. 35000835 10.1016/j.molmed.2021.12.002

[3] Kumar SR, Markusic DM, Biswas M, High KA, Herzog RW. Clinical development of gene therapy: results and lessons from recent successes. Mol Ther Methods Clin Dev. 2016;3:16034. 27257611 10.1038/mtm.2016.34 PMC4879992

[4] Bueren JA, Auricchio A. Advances and challenges in the development of gene therapy medicinal products for rare diseases. Hum Gene Ther. 2023;34:763–75. 37694572 10.1089/hum.2023.152

[5] Chancellor D, Barrett D, Nguyen-Jatkoe L, Millington S, Eckhardt F. The state of cell and gene therapy in 2023. Mol Ther. 2023;31:3376–88. 37927037 10.1016/j.ymthe.2023.11.001 PMC10727993

[6] Maguire AM, Bennett J, Aleman EM, Leroy BP, Aleman TS. Clinical perspective: treating RPE65-associated retinal dystrophy. Mol Ther. 2021;29:442–63. 33278565 10.1016/j.ymthe.2020.11.029 PMC7854308

[7] Bulcha JT, Wang Y, Ma H, Tai PWL, Gao G. Viral vector platforms within the gene therapy landscape. Signal Transduct Target Ther. 2021;6:53. 33558455 10.1038/s41392-021-00487-6 PMC7868676

[8] Strauss KA, Farrar MA, Muntoni F, Saito K, Mendell JR, Servais L, McMillan HJ, Finkel RS, Swoboda KJ, Kwon JM, Zaidman CM, Chiriboga CA, Iannaccone ST, Krueger JM, Parsons JA, Shieh PB, Kavanagh S, Tauscher-Wisniewski S, McGill BE, Macek TA. Onasemnogene abeparvovec for presymptomatic infants with two copies of SMN2 at risk for spinal muscular atrophy type 1: the Phase III SPR1NT trial. Nat Med. 2022;28:1381–9. 35715566 10.1038/s41591-022-01866-4 PMC9205281

[9] Strauss KA, Farrar MA, Muntoni F, Saito K, Mendell JR, Servais L, McMillan HJ, Finkel RS, Swoboda KJ, Kwon JM, Zaidman CM, Chiriboga CA, Iannaccone ST, Krueger JM, Parsons JA, Shieh PB, Kavanagh S, Wigderson M, Tauscher-Wisniewski S, McGill BE, Macek TA. Onasemnogene abeparvovec for presymptomatic infants with three copies of SMN2 at risk for spinal muscular atrophy: the Phase III SPR1NT trial. Nat Med. 2022;28:1390–7. 35715567 10.1038/s41591-022-01867-3 PMC9205287

[10] Locatelli F, Thompson AA, Kwiatkowski JL, Porter JB, Thrasher AJ, Hongeng S, Sauer MG, Thuret I, Lal A, Algeri M, Schneiderman J, Olson TS, Carpenter B, Amrolia PJ, Anurathapan U, Schambach A, Chabannon C, Schmidt M, Labik I, Elliot H, Guo R, Asmal M, Colvin RA, Walters MC. Betibeglogene autotemcel gene therapy for non-beta(0)/beta(0) genotype beta-thalassemia. N Engl J Med. 2022;386:415–27. 34891223 10.1056/NEJMoa2113206

[11] Bougneres P, Hacein-Bey-Abina S, Labik I, Adamsbaum C, Castaignede C, Bellesme C, Schmidt M. Long-term follow-up of hematopoietic stem-cell gene therapy for cerebral adrenoleukodystrophy. Hum Gene Ther. 2021;32:1260–9. 33789438 10.1089/hum.2021.053

[12] Eichler F, Duncan C, Musolino PL, Orchard PJ, De Oliveira S, Thrasher AJ, Armant M, Dansereau C, Lund TC, Miller WP, Raymond GV, Sankar R, Shah AJ, Sevin C, Gaspar HB, Gissen P, Amartino H, Bratkovic D, Smith NJC, Paker AM, Shamir E, O’Meara T, Davidson D, Aubourg P, Williams DA. Hematopoietic stem-cell gene therapy for cerebral adrenoleukodystrophy. N Engl J Med. 2017;377:1630–8. 28976817 10.1056/NEJMoa1700554 PMC5708849

[13] Fumagalli F, Calbi V, Natali Sora MG, Sessa M, Baldoli C, Rancoita PMV, Ciotti F, Sarzana M, Fraschini M, Zambon AA, Acquati S, Redaelli D, Attanasio V, Miglietta S, De Mattia F, Barzaghi F, Ferrua F, Migliavacca M, Tucci F, Gallo V, Del Carro U, Canale S, Spiga I, Lorioli L, Recupero S, Fratini ES, Morena F, Silvani P, Calvi MR, Facchini M, Locatelli S, Corti A, Zancan S, Antonioli G, Farinelli G, Gabaldo M, Garcia-Segovia J, Schwab LC, Downey GF, Filippi M, Cicalese MP, Martino S, Di Serio C, Ciceri F, Bernardo ME, Naldini L, Biffi A, Aiuti A. Lentiviral haematopoietic stem-cell gene therapy for early-onset metachromatic leukodystrophy: long-term results from a non-randomised, open-label, phase 1/2 trial and expanded access. Lancet. 2022;399:372–83. 35065785 10.1016/S0140-6736(21)02017-1 PMC8795071

[14] Ozelo MC, Mahlangu J, Pasi KJ, Giermasz A, Leavitt AD, Laffan M, Symington E, Quon DV, Wang JD, Peerlinck K, Pipe SW, Madan B, Key NS, Pierce GF, O’Mahony B, Kaczmarek R, Henshaw J, Lawal A, Jayaram K, Huang M, Yang X, Wong WY, Kim B. Valoctocogene roxaparvovec gene therapy for hemophilia A. N Engl J Med. 2022;386:1013–25. 35294811 10.1056/NEJMoa2113708

[15] Pipe SW, Leebeek FWG, Recht M, Key NS, Castaman G, Miesbach W, Lattimore S, Peerlinck K, Van der Valk P, Coppens M, Kampmann P, Meijer K, O’Connell N, Pasi KJ, Hart DP, Kazmi R, Astermark J, Hermans C, Klamroth R, Lemons R, Visweshwar N, von Drygalski A, Young G, Crary SE, Escobar M, Gomez E, Kruse-Jarres R, Quon DV, Symington E, Wang M, Wheeler AP, Gut R, Liu YP, Dolmetsch RE, Cooper DL, Li Y, Goldstein B, Monahan PE. Gene therapy with etranacogene dezaparvovec for hemophilia B. N Engl J Med. 2023;388:706–18. 36812434 10.1056/NEJMoa2211644

[16] Epstein AL, Haag-Molkenteller C. Herpes simplex virus gene therapy for dystrophic epidermolysis bullosa (DEB). Cell. 2023;186(3523–3523): e3521. 10.1016/j.cell.2023.07.031 37595560

[17] Kanter J, Walters MC, Krishnamurti L, Mapara MY, Kwiatkowski JL, Rifkin-Zenenberg S, Aygun B, Kasow KA, Pierciey FJ Jr, Bonner M, Miller A, Zhang X, Lynch J, Kim D, Ribeil JA, Asmal M, Goyal S, Thompson AA, Tisdale JF. Biologic and clinical efficacy of lentiglobin for sickle cell disease. N Engl J Med. 2022;386:617–28. 34898139 10.1056/NEJMoa2117175

[18] Frangoul H, Altshuler D, Cappellini MD, Chen YS, Domm J, Eustace BK, Foell J, de la Fuente J, Grupp S, Handgretinger R, Ho TW, Kattamis A, Kernytsky A, Lekstrom-Himes J, Li AM, Locatelli F, Mapara MY, de Montalembert M, Rondelli D, Sharma A, Sheth S, Soni S, Steinberg MH, Wall D, Yen A, Corbacioglu S. CRISPR-Cas9 gene editing for sickle cell disease and beta-thalassemia. N Engl J Med. 2021;384:252–60. 33283989 10.1056/NEJMoa2031054

[19] Cappelluti MA, Mollica Poeta V, Valsoni S, Quarato P, Merlin S, Merelli I, Lombardo A. Durable and efficient gene silencing in vivo by hit-and-run epigenome editing. Nature. 2024;627:416–23. 38418872 10.1038/s41586-024-07087-8 PMC10937395

[20] Watts JK, Corey DR. Silencing disease genes in the laboratory and the clinic. J Pathol. 2012;226:365–79. 22069063 10.1002/path.2993 PMC3916955

[21] Koike H, Katsuno M. Transthyretin amyloidosis: update on the clinical spectrum, pathogenesis, and disease-modifying therapies. Neurol Ther. 2020;9:317–33. 32948978 10.1007/s40120-020-00210-7 PMC7500251

[22] Ioannou A, Fontana M, Gillmore JD. RNA targeting and gene editing strategies for transthyretin amyloidosis. BioDrugs. 2023;37:127–42. 36795354 10.1007/s40259-023-00577-7 PMC9933836

[23] Zhang L, Lee C, Arnaoutova I, Anduaga J, Starost MF, Mansfield BC, Chou JY. Gene therapy using a novel G6PC-S298C variant enhances the long-term efficacy for treating glycogen storage disease type Ia. Biochem Biophys Res Commun. 2020;527:824–30. 32430177 10.1016/j.bbrc.2020.04.124 PMC7309276

[24] Smith EC, Hopkins S, Case LE, Xu M, Walters C, Dearmey S, Han SO, Spears TG, Chichester JA, Bossen EH, Hornik CP, Cohen JL, Bali D, Kishnani PS, Koeberl DD. Phase I study of liver depot gene therapy in late-onset Pompe disease. Mol Ther. 2023;31:1994–2004. 36805083 10.1016/j.ymthe.2023.02.014 PMC10362382

[25] Corti M, Liberati C, Smith BK, Lawson LA, Tuna IS, Conlon TJ, Coleman KE, Islam S, Herzog RW, Fuller DD, Collins SW, Byrne BJ. Safety of intradiaphragmatic delivery of adeno-associated virus-mediated alpha-glucosidase (rAAV1-CMV-hGAA) gene therapy in children affected by Pompe disease. Hum Gene Ther Clin Dev. 2017;28:208–18. 29160099 10.1089/humc.2017.146 PMC5733674

[26] Parker MA, Erker LR, Audo I, Choi D, Mohand-Said S, Sestakauskas K, Benoit P, Appelqvist T, Krahmer M, Segaut-Prevost C, Lujan BJ, Faridi A, Chegarnov EN, Steinkamp PN, Ku C, da Palma MM, Barale PO, Ayelo-Scheer S, Lauer A, Stout T, Wilson DJ, Weleber RG, Pennesi ME, Sahel JA, Yang P. Three-year safety results of SAR422459 (EIAV-ABCA4) gene therapy in patients with ABCA4-associated stargardt disease: an open-label dose-escalation phase I/IIa clinical trial, cohorts 1–5. Am J Ophthalmol. 2022;240:285–301. 35248547 10.1016/j.ajo.2022.02.013 PMC9308722

[27] Suarez-Herrera N, Li CHZ, Leijsten N, Karjosukarso DW, Corradi Z, Bukkems F, Duijkers L, Cremers FPM, Hoyng CB, Garanto A, Collin RWJ. Preclinical development of antisense oligonucleotides to rescue aberrant splicing caused by an ultrarare ABCA4 variant in a child with early-onset stargardt disease. Cells. 2024;13:601. 38607040 10.3390/cells13070601 PMC11011354

[28] McNamee SM, Chan NP, Akula M, Avola MO, Whalen M, Nystuen K, Singh P, Upadhyay AK, DeAngelis MM, Haider NB. Preclinical dose response study shows NR2E3 can attenuate retinal degeneration in the retinitis pigmentosa mouse model Rho(P23H+/)(). Gene Ther. 2024;31:255–62. 38273095 10.1038/s41434-024-00440-6 PMC11090815

[29] Lewis BS. First-in-human trial of PCSK9 gene editing therapy for lowering cholesterol: a new frontier in cardiovascular pharmacotherapy? News from AHA. Eur Heart J Cardiovasc Pharmacother. 2024;10:87–8. 38031331 10.1093/ehjcvp/pvad095

[30] Wilton-Clark H, Yokota T. Recent trends in antisense therapies for Duchenne muscular dystrophy. Pharmaceutics. 2023;15:778. 36986639 10.3390/pharmaceutics15030778 PMC10054484

[31] D’Ambrosio ES, Mendell JR. Evolving therapeutic options for the treatment of duchenne muscular dystrophy. Neurotherapeutics. 2023;20:1669–81. 37673849 10.1007/s13311-023-01423-y PMC10684843

[32] Mitelman O, Abdel-Hamid HZ, Byrne BJ, Connolly AM, Heydemann P, Proud C, Shieh PB, Wagner KR, Dugar A, Santra S, Signorovitch J, Goemans N, investigators from the, L. N. H. s., McDonald, C. M., investigators from the, C. D. N. H. S., Mercuri, E., investigators from The, D. M. D. I. G., and Mendell, J. R. A combined prospective and retrospective comparison of long-term functional outcomes suggests delayed loss of ambulation and pulmonary decline with long-term eteplirsen treatment. J Neuromuscul Dis. 2022;9:39–52. 34420980 10.3233/JND-210665 PMC8842766

[33] Scaglioni D, Catapano F, Ellis M, Torelli S, Chambers D, Feng L, Beck M, Sewry C, Monforte M, Harriman S, Koenig E, Malhotra J, Popplewell L, Guglieri M, Straub V, Mercuri E, Servais L, Phadke R, Morgan J, Muntoni F. The administration of antisense oligonucleotide golodirsen reduces pathological regeneration in patients with Duchenne muscular dystrophy. Acta Neuropathol Commun. 2021;9:7. 33407808 10.1186/s40478-020-01106-1 PMC7789286

[34] Mercuri E, Muntoni F, Osorio AN, Tulinius M, Buccella F, Morgenroth LP, Gordish-Dressman H, Jiang J, Trifillis P, Zhu J, Kristensen A, Santos CL, Henricson EK, McDonald CM, Desguerre I, Stride, and Investigators, C. D. N. H. Safety and effectiveness of ataluren: comparison of results from the STRIDE Registry and CINRG DMD Natural History Study. J Comp Eff Res. 2020;9:341–60. 31997646 10.2217/cer-2019-0171 PMC7610147

[35] Roberts TC. Long-term dystrophin restoration supports development of splice correction therapy for DMD patients with exon 2 duplications. Mol Ther Methods Clin Dev. 2023;31: 101160. 38074414 10.1016/j.omtm.2023.101160 PMC10709182

[36] Saade J, Mestre TA. Huntington’s disease: latest frontiers in therapeutics. Curr Neurol Neurosci Rep. 2024;24:255–64. 38861215 10.1007/s11910-024-01345-y

[37] Rook ME, Southwell AL. Antisense oligonucleotide therapy: from design to the Huntington disease clinic. BioDrugs. 2022;36:105–19. 35254632 10.1007/s40259-022-00519-9 PMC8899000

[38] Southwell AL, Kordasiewicz HB, Langbehn D, Skotte NH, Parsons MP, Villanueva EB, Caron NS, Ostergaard ME, Anderson LM, Xie Y, Cengio LD, Findlay-Black H, Doty CN, Fitsimmons B, Swayze EE, Seth PP, Raymond LA, Frank Bennett C, Hayden MR. Huntingtin suppression restores cognitive function in a mouse model of Huntington’s disease. Sci Transl Med. 2018;10:eaar3959. 30282695 10.1126/scitranslmed.aar3959

[39] Caron NS, Aly AE, Findlay Black H, Martin DDO, Schmidt ME, Ko S, Anderson C, Harvey EM, Casal LL, Anderson LM, Rahavi SMR, Reid GSD, Oda MN, Stanimirovic D, Abulrob A, McBride JL, Leavitt BR, Hayden MR. Systemic delivery of mutant huntingtin lowering antisense oligonucleotides to the brain using apolipoprotein A-I nanodisks for Huntington disease. J Control Release. 2024;367:27–44. 38215984 10.1016/j.jconrel.2024.01.011

[40] Caron NS, Southwell AL, Brouwers CC, Cengio LD, Xie Y, Black HF, Anderson LM, Ko S, Zhu X, van Deventer SJ, Evers MM, Konstantinova P, Hayden MR. Potent and sustained huntingtin lowering via AAV5 encoding miRNA preserves striatal volume and cognitive function in a humanized mouse model of Huntington disease. Nucleic Acids Res. 2020;48:36–54. 31745548 10.1093/nar/gkz976 PMC7145682

[41] Keller CG, Shin Y, Monteys AM, Renaud N, Beibel M, Teider N, Peters T, Faller T, St-Cyr S, Knehr J, Roma G, Reyes A, Hild M, Lukashev D, Theil D, Dales N, Cha JH, Borowsky B, Dolmetsch R, Davidson BL, Sivasankaran R. An orally available, brain penetrant, small molecule lowers huntingtin levels by enhancing pseudoexon inclusion. Nat Commun. 2022;13:1150. 35241644 10.1038/s41467-022-28653-6 PMC8894458

[42] Birolini G, Verlengia G, Talpo F, Maniezzi C, Zentilin L, Giacca M, Conforti P, Cordiglieri C, Caccia C, Leoni V, Taroni F, Biella G, Simonato M, Cattaneo E, Valenza M. SREBP2 gene therapy targeting striatal astrocytes ameliorates Huntington’s disease phenotypes. Brain. 2021;144:3175–90. 33974044 10.1093/brain/awab186

[43] Joy R, Phair K, O’Hara R, Brady D. Recent advances and current challenges in CAR-T cell therapy. Biotechnol Lett. 2024;46:115–26. 38150098 10.1007/s10529-023-03461-0

[44] Hsu RH, Hwu WL, Chen M, Chung IF, Peng SS, Chen CY, Cheng WC, Chien YH, Lee NC. Next-generation sequencing identifies TRPV4-related skeletal dysplasia in a boy with progressive bowlegs. Pediatr Neonatol. 2019;60:102–4. 29776788 10.1016/j.pedneo.2018.04.002

[45] Tai CH, Lee NC, Chien YH, Byrne BJ, Muramatsu SI, Tseng SH, Hwu WL. Long-term efficacy and safety of eladocagene exuparvovec in patients with AADC deficiency. Mol Ther. 2022;30:509–18. 34763085 10.1016/j.ymthe.2021.11.005 PMC8822132

[46] Naldini L, Trono D, Verma IM. Lentiviral vectors, two decades later. Science. 2016;353:1101–2. 27609877 10.1126/science.aah6192

[47] Cavazzana M, Bushman FD, Miccio A, Andre-Schmutz I, Six E. Gene therapy targeting haematopoietic stem cells for inherited diseases: progress and challenges. Nat Rev Drug Discov. 2019;18:447–62. 30858502 10.1038/s41573-019-0020-9

[48] Guffon N, Pettazzoni M, Pangaud N, Garin C, Lina-Granade G, Plault C, Mottolese C, Froissart R, Fouilhoux A. Long term disease burden post-transplantation: three decades of observations in 25 Hurler patients successfully treated with hematopoietic stem cell transplantation (HSCT). Orphanet J Rare Dis. 2021;16:60. 33517895 10.1186/s13023-020-01644-w PMC7847591

[49] Sailor KA, Agoranos G, Lopez-Manzaneda S, Tada S, Gillet-Legrand B, Guerinot C, Masson JB, Vestergaard CL, Bonner M, Gagnidze K, Veres G, Lledo PM, Cartier N. Hematopoietic stem cell transplantation chemotherapy causes microglia senescence and peripheral macrophage engraftment in the brain. Nat Med. 2022;28:517–27. 35190726 10.1038/s41591-022-01691-9

[50] Kida S, Koshimura Y, Yoden E, Yoshioka A, Morimoto H, Imakiire A, Tanaka N, Tanaka S, Mori A, Ito J, Inoue A, Yamamoto R, Minami K, Hirato T, Takahashi K, Sonoda H. Enzyme replacement with transferrin receptor-targeted alpha-L-iduronidase rescues brain pathology in mucopolysaccharidosis I mice. Mol Ther Methods Clin Dev. 2023;29:439–49. 37251981 10.1016/j.omtm.2023.05.010 PMC10220318

[51] Bradbury AM. Brain-targeted ex vivo lentiviral gene therapy: implications for MPS and beyond. Mol Ther Methods Clin Dev. 2023;31: 101137. 38027070 10.1016/j.omtm.2023.101137 PMC10630102

[52] Uchiyama T, Kawai T, Nakabayashi K, Nakazawa Y, Goto F, Okamura K, Nishimura T, Kato K, Watanabe N, Miura A, Yasuda T, Ando Y, Minegishi T, Edasawa K, Shimura M, Akiba Y, Sato-Otsubo A, Mizukami T, Kato M, Akashi K, Nunoi H, Onodera M. Myelodysplasia after clonal hematopoiesis with APOBEC3-mediated CYBB inactivation in retroviral gene therapy for X-CGD. Mol Ther. 2023;31:3424–40. 37705244 10.1016/j.ymthe.2023.09.004 PMC10727956

[53] Finkel RS, Darras BT, Mendell JR, Day JW, Kuntz NL, Connolly AM, Zaidman CM, Crawford TO, Butterfield RJ, Shieh PB, Tennekoon G, Brandsema JF, Iannaccone ST, Shoffner J, Kavanagh S, Macek TA, Tauscher-Wisniewski S. Intrathecal onasemnogene abeparvovec for sitting, nonambulatory patients with spinal muscular atrophy: phase I ascending-dose study (STRONG). J Neuromuscul Dis. 2023;10:389–404. 36911944 10.3233/JND-221560 PMC10200150

[54] Duan D. Lethal immunotoxicity in high-dose systemic AAV therapy. Mol Ther. 2023;31:3123–6. 37822079 10.1016/j.ymthe.2023.10.015 PMC10638066

[55] Samelson-Jones BJ, George LA. Adeno-associated virus gene therapy for hemophilia. Annu Rev Med. 2023;74:231–47. 36103998 10.1146/annurev-med-043021-033013 PMC9892335

[56] Chandler RJ, Di Pasquale G, Sloan JL, McCoy S, Hubbard BT, Kilts TM, Manoli I, Chiorini JA, Venditti CP. Systemic gene therapy for methylmalonic acidemia using the novel adeno-associated viral vector 44.9. Mol Ther Methods Clin Dev. 2022;27:61–72. 36186952 10.1016/j.omtm.2022.09.001 PMC9490190

[57] Han SO, Gheorghiu D, Li S, Kang HR, Koeberl D. Minimum effective dose to achieve biochemical correction with adeno-associated virus vector-mediated gene therapy in mice with pompe disease. Hum Gene Ther. 2022;33:492–8. 35102744 10.1089/hum.2021.252

[58] Shirley JL, de Jong YP, Terhorst C, Herzog RW. Immune responses to viral gene therapy vectors. Mol Ther. 2020;28:709–22. 31968213 10.1016/j.ymthe.2020.01.001 PMC7054714

[59] George LA, Monahan PE, Eyster ME, Sullivan SK, Ragni MV, Croteau SE, Rasko JEJ, Recht M, Samelson-Jones BJ, MacDougall A, Jaworski K, Noble R, Curran M, Kuranda K, Mingozzi F, Chang T, Reape KZ, Anguela XM, High KA. Multiyear factor VIII expression after AAV gene transfer for hemophilia A. N Engl J Med. 2021;385:1961–73. 34788507 10.1056/NEJMoa2104205 PMC8672712

[60] Dalwadi DA, Calabria A, Tiyaboonchai A, Posey J, Naugler WE, Montini E, Grompe M. AAV integration in human hepatocytes. Mol Ther. 2021;29:2898–909. 34461297 10.1016/j.ymthe.2021.08.031 PMC8531150

[61] Tabebordbar M, Lagerborg KA, Stanton A, King EM, Ye S, Tellez L, Krunnfusz A, Tavakoli S, Widrick JJ, Messemer KA, Troiano EC, Moghadaszadeh B, Peacker BL, Leacock KA, Horwitz N, Beggs AH, Wagers AJ, Sabeti PC. Directed evolution of a family of AAV capsid variants enabling potent muscle-directed gene delivery across species. Cell. 2021;184(4919–4938): e4922. 10.1016/j.cell.2021.08.028 PMC9344975 34506722

[62] Huang Q, Chan KY, Wu J, Botticello-Romero NR, Zheng Q, Lou S, Keyes C, Svanbergsson A, Johnston J, Mills A, Lin CY, Brauer PP, Clouse G, Pacouret S, Harvey JW, Beddow T, Hurley JK, Tobey IG, Powell M, Chen AT, Barry AJ, Eid FE, Chan YA, Deverman BE. An AAV capsid reprogrammed to bind human transferrin receptor mediates brain-wide gene delivery. Science. 2024;384:eadm8386. 10.1126/science.adm8386 PMC13387621 38753766

[63] Crane R, Conley SM, Al-Ubaidi MR, Naash MI. Gene therapy to the retina and the cochlea. Front Neurosci. 2021;15: 652215. 33815052 10.3389/fnins.2021.652215 PMC8010260

[64] Rotstein M, Kang UJ. Consideration of gene therapy for paediatric neurotransmitter diseases. J Inherit Metab Dis. 2009;32:387–94. 19259783 10.1007/s10545-009-1054-7 PMC4848069

[65] Iankova V, Karin I, Klopstock T, Schneider SA. Emerging disease-modifying therapies in neurodegeneration with brain iron accumulation (NBIA) disorders. Front Neurol. 2021;12: 629414. 33935938 10.3389/fneur.2021.629414 PMC8082061

[66] Lange LM, Junker J, Loens S, Baumann H, Olschewski L, Schaake S, Madoev H, Petkovic S, Kuhnke N, Kasten M, Westenberger A, Domingo A, Marras C, Konig IR, Camargos S, Ozelius LJ, Klein C, Lohmann K. Genotype-phenotype relations for isolated dystonia genes: MDSGene systematic review. Mov Disord. 2021;36:1086–103. 33502045 10.1002/mds.28485

[67] Yamazaki Y, Hirai Y, Miyake K, Shimada T. Targeted gene transfer into ependymal cells through intraventricular injection of AAV1 vector and long-term enzyme replacement via the CSF. Sci Rep. 2014;4:5506. 24981028 10.1038/srep05506 PMC4076682

[68] Chen X, Lim DA, Lawlor MW, Dimmock D, Vite CH, Lester T, Tavakkoli F, Sadhu C, Prasad S, Gray SJ. Biodistribution of adeno-associated virus gene therapy following cerebrospinal fluid-directed administration. Hum Gene Ther. 2023;34:94–111. 36606687 10.1089/hum.2022.163

[69] Marco S, Haurigot V, Jaen ML, Ribera A, Sanchez V, Molas M, Garcia M, Leon X, Roca C, Sanchez X, Bertolin J, Perez J, Elias G, Navarro M, Carretero A, Pumarola M, Andaluz A, Espada Y, Anor S, Bosch F. Seven-year follow-up of durability and safety of AAV CNS gene therapy for a lysosomal storage disorder in a large animal. Mol Ther Methods Clin Dev. 2021;23:370–89. 34761052 10.1016/j.omtm.2021.09.017 PMC8550992

[70] Iwakawa HO, Tomari Y. Life of RISC: formation, action, and degradation of RNA-induced silencing complex. Mol Cell. 2022;82:30–43. 34942118 10.1016/j.molcel.2021.11.026

[71] Eckstein F. Phosphorothioates, essential components of therapeutic oligonucleotides. Nucleic Acid Ther. 2014;24:374–87. 25353652 10.1089/nat.2014.0506

[72] Khvorova A, Watts JK. The chemical evolution of oligonucleotide therapies of clinical utility. Nat Biotechnol. 2017;35:238–48. 28244990 10.1038/nbt.3765 PMC5517098

[73] Ventura P, Bonkovsky HL, Gouya L, Aguilera-Peiro P, Montgomery Bissell D, Stein PE, Balwani M, Anderson DKE, Parker C, Kuter DJ, Monroy S, Oh J, Ritchie B, Ko JJ, Hua Z, Sweetser MT, Sardh E, Investigators E. Efficacy and safety of givosiran for acute hepatic porphyria: 24-month interim analysis of the randomized phase 3 ENVISION study. Liver Int. 2022;42:161–72. 34717041 10.1111/liv.15090 PMC9299194

[74] Finkel RS, Mercuri E, Darras BT, Connolly AM, Kuntz NL, Kirschner J, Chiriboga CA, Saito K, Servais L, Tizzano E, Topaloglu H, Tulinius M, Montes J, Glanzman AM, Bishop K, Zhong ZJ, Gheuens S, Bennett CF, Schneider E, Farwell W, De Vivo DC. Nusinersen versus sham control in infantile-onset spinal muscular atrophy. N Engl J Med. 2017;377:1723–32. 29091570 10.1056/NEJMoa1702752

[75] Crooke ST, Baker BF, Kwoh TJ, Cheng W, Schulz DJ, Xia S, Salgado N, Bui HH, Hart CE, Burel SA, Younis HS, Geary RS, Henry SP, Bhanot S. Integrated safety assessment of 2’-O-methoxyethyl chimeric antisense oligonucleotides in nonhuman primates and healthy human volunteers. Mol Ther. 2016;24:1771–82. 27357629 10.1038/mt.2016.136 PMC5112040

[76] Crooke ST. Addressing the needs of patients with ultra-rare mutations one patient at a time: the n-lorem approach. Nucleic Acid Ther. 2022;32:95–100. 34520268 10.1089/nat.2021.0046

[77] Kim J, Hu C, Moufawad El Achkar C, Black LE, Douville J, Larson A, Pendergast MK, Goldkind SF, Lee EA, Kuniholm A, Soucy A, Vaze J, Belur NR, Fredriksen K, Stojkovska I, Tsytsykova A, Armant M, DiDonato RL, Choi J, Cornelissen L, Pereira LM, Augustine EF, Genetti CA, Dies K, Barton B, Williams L, Goodlett BD, Riley BL, Pasternak A, Berry ER, Pflock KA, Chu S, Reed C, Tyndall K, Agrawal PB, Beggs AH, Grant PE, Urion DK, Snyder RO, Waisbren SE, Poduri A, Park PJ, Patterson A, Biffi A, Mazzulli JR, Bodamer O, Berde CB, Yu TW. Patient-customized oligonucleotide therapy for a rare genetic disease. N Engl J Med. 2019;381:1644–52. 31597037 10.1056/NEJMoa1813279 PMC6961983

[78] Lauffer MC, van Roon-Mom W, Aartsma-Rus A, Collaborative N. Possibilities and limitations of antisense oligonucleotide therapies for the treatment of monogenic disorders. Commun Med (Lond). 2024;4:6. 38182878 10.1038/s43856-023-00419-1 PMC10770028

[79] Hillary VE, Ceasar SA. A review on the mechanism and applications of CRISPR/Cas9/Cas12/Cas13/Cas14 proteins utilized for genome engineering. Mol Biotechnol. 2023;65:311–25. 36163606 10.1007/s12033-022-00567-0 PMC9512960

[80] Porto EM, Komor AC. In the business of base editors: evolution from bench to bedside. PLoS Biol. 2023;21: e3002071. 37043430 10.1371/journal.pbio.3002071 PMC10096463

[81] Li A, Mitsunobu H, Yoshioka S, Suzuki T, Kondo A, Nishida K. Cytosine base editing systems with minimized off-target effect and molecular size. Nat Commun. 2022;13:4531. 35941130 10.1038/s41467-022-32157-8 PMC9359979

[82] Bhatia S, Le Cam Y, Carrion J, Diamond L, Fennessy P, Gassman S, Gutzwiller F, Kagan S, Pankevich D, Young Maloney J, Mahadev N, Schulz M, Wong-Rieger D, Morgese P. Strengthening health systems for access to gene therapy in rare genetic disorders. Mol Ther Methods Clin Dev. 2024;32: 101220. 38516694 10.1016/j.omtm.2024.101220 PMC10951444

[83] Wong CH, Li D, Wang N, Gruber J, Lo AW, Conti RM. The estimated annual financial impact of gene therapy in the United States. Gene Ther. 2023;30:761–73. 37935855 10.1038/s41434-023-00419-9 PMC10678302

[84] Montazerhodjat V, Weinstock DM, Lo AW. Buying cures versus renting health: financing health care with consumer loans. Sci Transl Med. 2016;8:327ps326. 10.1126/scitranslmed.aad6913 26912902

[85] Booth C, Aiuti A. Realizing the potential of gene therapies for rare and ultra-rare inherited diseases. Hum Gene Ther. 2023;34:776–81. 37624746 10.1089/hum.2023.127

[86] Das S, Huang S, Lo AW. Acceleration of rare disease therapeutic development: a case study of AGIL-AADC. Drug Discov Today. 2019;24:678–84. 30610920 10.1016/j.drudis.2018.12.006

